# Roxadustat Attenuates Adverse Remodeling Following Myocardial Infarction in Mice

**DOI:** 10.3390/cells13131074

**Published:** 2024-06-21

**Authors:** Marc-Michael Zaruba, Simon Staggl, Santhosh Kumar Ghadge, Thomas Maurer, Jasmina Gavranovic-Novakovic, Vivek Jeyakumar, Patric Schönherr, Andreas Wimmer, Gerhard Pölzl, Axel Bauer, Moritz Messner

**Affiliations:** 1 Department of Internal Medicine III, Cardiology and Angiology, Medical University Innsbruck, 6020 Innsbruck, Austria; marc-michael.zaruba@i-med.ac.at (M.-M.Z.); simon.staggl@tirol-kliniken.at (S.S.); santhoshkumar.ghadge@valneva.com (S.K.G.); thomas.maurer@i-med.ac.at (T.M.); jasmina.gavranovich@i-med.ac.at (J.G.-N.); patric.schoenherr@student.i-med.ac.at (P.S.); gerhard.poelzl@tirol-kliniken.at (G.P.); axel.bauer@i-med.ac.at (A.B.); 2 Valneva Austria GmbH, Campus Vienna Biocenter 3, 1030 Vienna, Austria; 3 Department of Surgery, Kardinal Schwarzenberg Klinikum GmbH, 5620 Salzburg, Austria; andreas.wimmer@ksk-klinikum.at

**Keywords:** prolyl hydroxylase inhibitor, hypoxia inducible factor-1, acute coronary syndrome, cardiomyopathy, adverse remodeling, chemokines, inflammation, fibrosis, apoptosis

## Abstract

Activation of the CXCL12/CXCR4/ACKR3 axis is known to aid myocardial repair through ischemia-triggered hypoxia-inducible factor-1α (HIF-1α). To enhance the upregulation of HIF-1α, we administered roxadustat, a novel prolyl hydroxylase inhibitor (PHI) clinically approved by the European Medicines Agency 2021 for the treatment of renal anemia, with the purpose of improving LV function and attenuating ischemic cardiomyopathy. Methods: We evaluated roxadustat’s impact on HIF-1 stimulation, cardiac remodeling, and function after MI. Therefore, we analyzed nuclear HIF-1 expression, the mRNA and protein expression of key HIF-1 target genes (RT-PCR, Western blot), inflammatory cell infiltration (immunohistochemistry), and apoptosis (TUNEL staining) 7 days after MI. Additionally, we performed echocardiography in male and female C57BL/6 mice 28 days post-MI. Results: We found a substantial increase in nuclear HIF-1, associated with an upregulation of HIF-1α target genes like CXCL12/CXCR4/ACKR3 at the mRNA and protein levels. Roxadustat increased the proportion of myocardial reparative M2 CD206+ cells, suggesting beneficial alterations in immune cell migration and a trend towards reduced apoptosis. Echocardiography showed that roxadustat treatment significantly preserved ejection fraction and attenuated subsequent ventricular dilatation, thereby reducing adverse remodeling. Conclusions: Our findings suggest that roxadustat is a promising clinically approved treatment option to preserve myocardial function by attenuating adverse remodeling.

## 1. Introduction

Ischemic heart disease following myocardial infarction (MI) often leads to heart failure (HF) associated with high rates of mortality and morbidity [1]. Advancements in medical treatments and interventional techniques have reduced mortality rates. However, with more patients surviving MI, the prevalence of subsequent HF due to ischemic cardiomyopathy is rising. Consequently, a considerable number of post-MI patients depend on heart transplantation or LVAD (left ventricular assistant device) implantation as their final treatment option. Therefore, the development of therapeutic approaches designed to improve myocardial function is warranted.

Prolyl hydroxylase inhibitors (PHIs) are a new type of drug class known for their ability to stabilize hypoxia-inducible factor (HIF) α-subunits, which are pivotal transcriptional orchestrators in the hypoxic response. They have the potential to upregulate reparative target genes like vascular endothelial growth factor (VEGF), erythropoietin (EPO), and C-X-C motif chemokine 12 (CXCL12) [2].

HIF-1α, upon dimerization with the β-subunit, translocates to the nucleus and binds to HIF-responsive elements. This initiates the transcription of many target genes essential for physiological processes such as erythropoiesis (EPO), angiogenesis (VEGF, CXCL12), energy metabolism, inflammation, cell survival, and extracellular matrix constitution [3,4,5].

In the context of myocardial infarction (MI), PHIs hold promise as a reparative strategy to attenuate adverse remodeling by increasing HIF-1α levels.

Central to myocardial repair is the CXCL12/CXCR4/ACKR3 axis, altogether targeting genes of HIF-1. The combined effects of C-X-C motif chemokine 12 (CXCL12) and its specific receptors, C-X-C motif chemokine receptor 4 (CXCR4) and atypical chemokine receptor 3 (ACKR3), along with other entities regulated by HIF-1, support processes such as cellular migration, cell survival, formation of new blood vessels, remodeling of the extra-cellular matrix, and regulation of inflammation. Together, these actions help to reduce the overall damage caused by MI and stimulate myocardial repair [6,7,8,9,10]. Previous studies have shown that HIF-1α can help preserve myocardial function and minimize scarring after myocardial infarction, and the toxic effects of some agents have prevented clinical translation so far [11].

To overcome this limitation, we focus on roxadustat, a PHI approved by the European Medicines Agency [12] in 2021 for treating anemia in individuals suffering from chronic kidney disease (CKD). Our research suggests that roxadustat could be an effective therapy for protecting cardiac tissue against hypoxemic injury because of its favorable safety profile.

This manuscript investigates the therapeutic potential of roxadustat in mitigating ischemic cardiomyopathy after MI in a C57BL/6 murine model. Recent studies have shown that CXCL12 enhances the angiogenic potential of human umbilical vein-derived endothelial cells (HUVECs) [13,14]. Hence, dosage optimization was performed in vitro on HUVECs to understand the molecular mechanisms of cardiac vascular morphogenesis and the interaction between endothelial cells and cardiomyocytes for creating effective therapies [15,16]. We propose that roxadustat is a promising treatment option by modulating HIF-1α and downstream molecular pathways, thereby attenuating adverse post-MI remodeling.

## 2. Materials and Methods

### 2.1. In Vitro Experiments with Roxadustat-Treated HUVECs

HUVECs were cultured in endothelial cell medium (ECM) supplemented with 10% fetal bovine serum, 1% endothelial cell growth supplement, and 1% penicillin–streptomycin. Cells were maintained in a humidified incubator at 37 °C with 5% CO_2_ and routinely passaged upon reaching 80–90% confluence.

### 2.2. Dosage Optimization for Treatment with Roxadustat

For dosage in vitro optimization, HUVECs were seeded on a T75 flask (Greiner Bio-One GmbH, Kremsmünster, Germany) and allowed to adhere overnight. At 80–90% confluency, the cells were treated with different concentrations of roxadustat (FG4592, Cayman Chemicals, Ann Arbor, MI, USA) (20, 50, 100, and 500 µM) dissolved in DMSO (Invitrogen, Thermo Fisher Scientific, Waltham, MA, USA). The control group was treated with 7.5 µM DMSO. After a 24-h incubation period, the cells were harvested for Western blot analysis to evaluate HIF-1α expression levels and determine the optimal roxadustat concentration for subsequent experiments.

After establishing the optimal roxadustat concentration, a time course study was initiated. HUVECs were treated with 100 µM roxadustat, and the cells were harvested and pooled from 6 replicates of a 6-well plate at various time points post-treatment (1, 2, 6, and 24 h) to monitor the temporal expression patterns of HIF-1α.

### 2.3. Animal Housing

Male and female C57BL/6 mice (Charles River Laboratories) were housed in a controlled environment in compliance with Austrian and European regulations, specifically, Directive 2010/63/EU on the protection of animals used for scientific purposes, and were approved by the Federal Ministry of Education, Science and Research (BMBWF) enforcing the Animal Testing Act 2012 (TVG 2012, GZ 2022-0.920.307, 17 January 2023). All efforts were made to minimize the suffering and the number of mice used.

The room temperature was maintained at 20–24 °C with a relative humidity of 40–60%. A 12-h light/dark cycle was implemented.

Mice were kept in Type II cages made of autoclavable polycarbonate, as per the European Convention for the Protection of Vertebrate Animals. Each cage was equipped with a stainless-steel wire lid and a water bottle. The bedding material was replaced at least once a week to maintain hygiene. Each cage housed a maximum of 5 mice to prevent overcrowding and to ensure social interaction among the animals.

In line with the 3Rs principle (Replacement, Reduction, Refinement), environmental enrichment was provided to enhance the well-being of the mice.

Mice were fed a standard rodent diet that met the nutritional requirements outlined in the European guidelines. Food pellets were available ad libitum, and the water bottles were filled with autoclaved tap water, also available ad libitum. The food and water supplies were checked daily and replenished as needed.

A health surveillance program was in place to monitor the health status of the mice. Veterinary staff conducted regular health checks and any signs of illness or distress were promptly addressed. All procedures were carried out under aseptic conditions to minimize the risk of infection. All efforts were made to minimize the number of animals and their suffering.

### 2.4. LAD-Ligation in Mice

Eight- to ten-week-old male and female C57BL/6 mice (Charles River Laboratories) were anesthetized using a mixture of ketamine (100 mg/kg) (Sigma Chemical Co., St. Louis, MO, USA) and xylazine (5 mg/kg) (Sigma Chemical Co., St. Louis, MO, USA), administered intraperitoneally (i.p.). After achieving adequate anesthesia and analgesia, the mice were placed in a supine position on a heated surgical pad to maintain body temperature. Hair from the chest area was removed with a commercially available hair-removing paste and disinfected using an antiseptic solution. The mice were provided with artificial ventilation via a mouse ventilator (Hugo Sachs Elektronik, Harvard Apparatus, March, Germany) at a rate of 180 strokes per minute and 180 µL per stroke.

A left anterolateral thoracotomy was performed to expose the heart, and the left anterior descending artery (LAD) was identified. An 8-0 Prolene suture (Ethicon, Johnson & Johnson, Somerville, NJ, USA) was used to ligate the LAD, thereby inducing myocardial ischemia. The chest was then closed in layers, and the skin was sutured using 6-0 Vicryl (Ethicon, Johnson & Johnson, Somerville, NJ, USA) sutures. During anesthesia, a single dose of carprofen (5 mg/kg) (Rimadyl, Pfizer, New York, NY, USA) was administered i.p. for preemptive analgesia. 

The mice were then returned to their home cages, where they were closely monitored for any signs of distress or complications and kept warm with infrared lamps until they fully recovered from anesthesia. The cages were placed on heating pads to help maintain body temperature during the initial recovery period.

Postoperatively, additional doses of carprofen (5 mg/kg) were given at 24-h intervals for the next three days to manage pain and inflammation. Furthermore, buprenorphine (Richter Pharma AG, Wels, Austria) was added to the drinking water at a concentration of 0.5 mg/L for 7 days post-surgery to provide sustained analgesia.

All surgical and analgesic procedures were approved by the Institutional Animal Care and Use Committee (IACUC) and were performed in strict accordance with Austrian and European regulations for animal welfare. Special attention was given to the 3Rs principle (Replacement, Reduction, Refinement) to minimize animal suffering. The animal studies were approved by the Austrian Federal Ministry of Education, Science and Research under the file number 2023-0.515.096.

### 2.5. Roxadustat Treatment

In this study, roxadustat was administered to C57BL/6 mice to investigate its effects under different experimental conditions. The administration route was intraperitoneal, utilizing a dosing regimen of 50 mg/kg body weight. This dosage was administered twice weekly. The duration of the treatment varied according to the specific experimental design, with mice receiving roxadustat for either 7 days or 28 days.

### 2.6. Echocardiographic Assessment

Echocardiographic measurements were performed 28 days post-LAD ligation using a high-frequency ultrasound Vevo 2100 system (FUJIFILM VisualSonics Inc., Toronto, ON, Canada) equipped with an 18–30-MHz linear array transducer. The heart was imaged in the parasternal long-axis and short-axis views. All measurements were performed by an experienced operator who was blinded to the experimental conditions.

The mice were anesthetized using isoflurane (1.5–2.0% in 100% oxygen, Baxter Inc., Deerfield, IL, USA) via a nose cone. The depth of anesthesia was monitored by the toe pinch reflex and respiratory rate. The animals were placed in a supine position on a self-regulating heated pad to maintain body temperature and prevent hypothermia. Hair over the chest area was removed using a commercial hair removal paste to improve the quality of the ultrasound images. 

End-diastolic and end-systolic volumes were measured using the Simpson’s method. The left ventricular (LV) cavity was traced at end-diastole and end-systole in the short axis view at three levels (basal, mid, and apical). The LV volumes were calculated by summing the volumes of the individual disks. The length of the left ventricle was determined in the parasternal long-axis view in systole and diastole, and longitudinal shortening was determined {[(length;d − length;s)/length;d] × 100}.

In M-mode echocardiography, we used the long-axis view to measure the following parameters: IVSd, LVEDD, and LVESD.

All data were analyzed using Vevo LAB 3.0.0 software (FUJIFILM VisualSonics Inc.) in a blinded fashion.

### 2.7. Western Blot

The PARIS Kit by Invitrogen (Invitrogen, Thermo Fisher Scientific, Waltham, MA, USA) was employed to extract protein from total heart tissue (in vivo). Briefly, cell fractionation buffer was added and centrifuged at 4 °C, cell disruption buffer was added to the pellet, and the resulting nuclear lysate was used for protein analysis. Thermo Fisher’s Pierce RIPA Lysis Buffer was used for protein isolation from the cultured cells (in vitro) according to the manufacturer’s protocol. The protein concentration was determined using the Pierce BCA Protein Assay (Invitrogen, Thermo Fisher Scientific). Equal amounts of protein were loaded into a 4–20% precast polyacrylamide gel (Bio-Rad Laboratories, Inc., Hercules, CA, USA) and subjected to electrophoresis. Following separation, the proteins were transferred onto a polyvinylidene fluoride (PVDF) Amersham Hybond p 0.45 PVDF membrane (GE Healthcare Life Sciences, Chicago, IL, USA) using a wet transfer system (Bio-Rad).

The membrane was blocked with 5% non-fat milk in TBST (Tris-buffered saline, 0.1% Tween 20) for 1 h at room temperature. Primary antibodies were applied and incubated overnight at 4 °C. After washing with TBST, the membrane was incubated with appropriate peroxidase-coupled secondary antibodies (1:5000) for 1 h at room temperature.

The membrane was washed three times with TBST and developed using ECL Prime (Amersham, Cytiva, Marlborough, MA, USA) according to the manufacturer’s guidelines. Chemiluminescence was detected by a ChemiDoc Imaging System (Bio-Rad Laboratories Inc.).

The captured images were analyzed using Bio-Rads ImageLab software v6.0 (Bio-Rad Laboratories Inc.) and Image J v1.54j (Wayne Rasband, National Institutes of Health, Bethesda, MD, USA) [17]. Band intensities were quantified and normalized to the housekeeping protein GAPDH or TBP to account for any variations in protein loading. The fold change was always calculated from the sample with the lowest expression within the control group.

Anti-GAPDH antibody (ab8245; Abcam, Cambridge, UK) (1:10,000).

Anti-TBP antibody (ab51841; Abcam) (1:1000).

Anti-HIF-1 alpha antibody (ab179483; Abcam) (1:1000).

Anti-CXCR4 antibody (ab124824; Abcam) (1:500).

Anti-GPCR RDC1/CXCR-7 antibody (ab138509; Abcam) (1:1000).

SDF1/CXCL12 antibody #3740 (Cell Signaling Technology, Boston, MA, USA) (1:1000).

### 2.8. Quantitative Real-Time PCR (qRT-PCR) Analysis

Total RNA was extracted from the collected tissue samples using TRI Reagent (Sigma Aldrich, Darmstadt, Germany) following the manufacturer’s instructions. The concentration and purity of the extracted RNA were determined using a NanoDrop spectrophotometer (Thermo Fischer Scientific, Munich, Germany). Complementary DNA (cDNA) was synthesized from 500 ng of total RNA using the QuantiTect Reverse Transcription Kit (QIAGEN, Hilden, Germany), following the manufacturer’s protocol. After transcription, cDNA was diluted with 170 µL H_2_O to a total volume of 190 µL.

Specific primers for the target genes and the reference gene were synthesized by Eurofins (Eurofins Genomics, Ebersberg, Germany). The sequences of the primers used are as follows:
CXCL12α Forward: 5′-ACACTCCAAACTGTGCCCTT-3′.CXCL12α Reverse: 5′-GCATCTCCCACGGATGTCAG-3′.CXCR4 Forward: 5′-CGGCTGTAGAGCGAGTGTTG-3′.CXCR4 Reverse: 5′-GCAGGGTTCCTTGTTGGAGT-3′.ACKR3 Forward:5′-CACCGTCAGGAAGGCAAACC-3′.ACKR3 Reverse: 5′-CAATGCAGTCGCTGCTGTTAC-3′.VEGFA Forward: 5′-GCACTGGACCCTGGCTTTAC-3′.VEGFA Reverse: 5′-GTCTCAATCGGACGGCAGTA-3′.VEGFD Forward: 5′-GAGCGAACATGGACCAGTGA-3′.VEGFD Reverse: 5′-GTCTCAATCGGACGGCAGTA-3′.Col1A1 Forward: 5′-GCTCCTCTTAGGGGCCACT-3′.Col1A1 Reverse: 5′-CCACGTCTCACCATTGGGG-3′.AMD Forward: 5′-TTCTCGGCTTCTCATCGCAG-3′.AMD Reverse:5′-TCTCATCAGCGAGTCCCGTA-3′.FGF2 Forward:5′-GGCTGCTGGCTTCTAAGTGT-3′.FGF2 Reverse:5′-GGTTTTGCCCTGGACCTGTC-3′.GLUT-1 Forward:5′-TTGAGTTGAGAAGCCCCGAC-3‘.GLUT-1 Reverse:5′-AGATCCCCACGCAAAGTCTG-3′.Col8A1 Forward:5′-CTCTTGGTCCAGGTTCTCCA-3′.Col8A1 Reverse:5′-AAGGAAATCCCACCTGTGC-3.RPL32 Forward: 5′-TCCTGGTCCACAATGTCAAG-3′.RPL32 Reverse: 5′-AGCTGTGCTGCTCTTTCTAC-3′.

Quantitative real-time PCR was performed using the PowerUp SYBR Green Master Mix (Applied Biosystems, Waltham, MA, USA) on a StepOnePlus Real-Time PCR System (Applied Biosystems). Each reaction mixture (20 µL) contained 10 µL of SYBR Green Master Mix, 0.25 µM of each primer, and 9.5 µL of cDNA template. The reactions were set up in duplicates.

The threshold cycle (Ct) values were determined using the instrument’s software, and the relative expression levels of the target genes were calculated using the 2^−ΔΔCt^ method [18]. The expression levels of the target genes were normalized to the reference gene Ribosomal Protein L32 (RPL32), and the fold change in mRNA expression was calculated relative to the sample with the lowest expression within the control group.

### 2.9. Immunohistochemical Staining

Whole hearts were obtained 7 days after MI and embedded in optimal cutting temperature (O.C.T.) compound (Sakura Finetek, Alphen aan den Rijn, The Netherlands). Cryosections were cut at a thickness of 4 µm and mounted on glass slides.

Cryosections were allowed to air-dry briefly and then fixed in cold acetone for 10 min. After fixation, sections were rehydrated in TBST. Endogenous peroxidase activity was quenched with Bloxall (Vector Laboratories, Burlingame, CA, USA) for 10 min.

Primary antibodies (Abcam, Cambridge, UK) were diluted in Dako Antibody Dilutand with Background Reducing Components (Dako, Carpinteria, CA, USA) and applied to tissue sections at the following dilutions: CD45 (1:400, ab33429), CD31 (1:200, ab281583), CD206 (1:500, ab300621), CD68 (1:4000, ab125212), vWF (1:200, ab287962), and CD3ε (1:1000, ab215212).

The sections were incubated with primary antibodies in a humidified chamber for one hour at room temperature (RT).

After primary antibody incubation, the sections were washed in TBST and incubated with the EnVision+ System-HRP Labelled Polymer Anti-Rabbit secondary antibody (Dako, Carpinteria, CA, USA) for 30 min at RT.

Secondary antibodies were applied and incubated for 30 min at RT.

Immunoreactivity was visualized using the Dako Liquid DAB+ Substrate Chromogen System for 10 min. The sections were counterstained with hematoxylin for one minute to visualize cell nuclei. After counterstaining, the slides were washed, dehydrated, cleared in xylene, and coverslipped with a mounting medium.

Stained tissue cryosections were examined using a NanoZoomer S210 (Hamamatsu Photonics, Shizuaka, Japan) at ×40 magnification and quantified using automated cell count (QuPath, V0.3.2., https://www.nature.com/articles/s41598-017-17204-5, accessed on 11 June 2024) in 4 high power fields (HPFs).

### 2.10. Apoptotic Index Measurement Using the Immunofluorescent TUNEL Assay

Whole heart sections were prepared as described above. The TUNEL assay was performed using the On-step TUNEL In Situ Apoptosis Kit Red Elab Fluor 647 (Elabscience Biotechnology Co., Ltd., Houston, TX, USA), according to the manufacturer’s instructions. Images were captured using an Olympus VS120 microscope (Olympus Corporation, Tokyo, Japan) equipped with a 40× objective lens.

To ensure the images were seamlessly processed, automated cell counting was performed by the Olympus CellSens software v4.3.

We looked for apoptotic hot spots in every sample and imaged 4 HPFs per sample. For each HPF, the number of DAPI-positive cells and the number of TUNEL-positive cells were counted. The apoptotic index for each field was calculated as the ratio of TUNEL-positive cells to the number of DAPI-positive cells, multiplied by 100 to obtain a percentage. The mean apoptotic index for each sample was then calculated by averaging the apoptotic indices of the four HPFs.

### 2.11. Statistical Analysis

Student’s *t*-test was performed for comparisons of two datasets at a time. All data are presented as the mean ± SD. The significance level was set at *p* < 0.05. All statistical analyses were performed using IBM SPSS Statistics (IBM Corp. Released 2023. IBM SPSS Statistics for Windows, Version 29.0.2.0 Armonk, NY, USA: IBM Corp). All graphs were created using the GraphPad Prism 9 software (Graphpad Prism Software Inc., San Diego, CA, USA).

## 3. Results

### 3.1. Roxadustat Effectively Activates Dose-Dependent HIF-1α Expression in HUVECs

**Figure 1:**
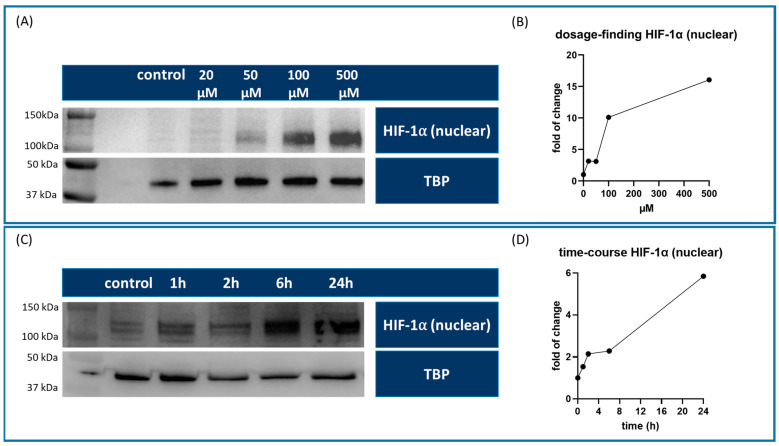
Western blots of the dosage optimization experiment in HUVEC cells. (**A**) Nuclear fraction of HIF-1α and TBP for loading control. (**B**) Nuclear HIF-1α normalized to TATA-binding protein (TBP) suggests a dose optimum at 100 µM. (**C**) Nuclear HIF-1α and TBP for loading control. (**D**) Nuclear HIF-1α expression shows a steady rise over time and reaches its maximum after 24 h.

First, we evaluated the dose-dependent effect of roxadustat on the expression of nuclear HIF-1α in human umbilical vein endothelial cells (HUVECs). Therefore, the HUVECs were exposed to increasing concentrations of roxadustat (FG-4592, Cayman Chemicals, Ann Arbor, MI, USA) (20, 50, 100, and 500 µM), while the control cells were incubated with 7.5 µM dimethyl sulfoxide (DMSO). Western blot analyses were performed from six pooled replicates, and the densitometric measurements showed that the expression of nuclear HIF-1α protein levels increased with increasing concentrations of roxadustat in a dose-dependent manner. At lower concentrations (20 and 50 µM), the increase in HIF-1α expression was modest, while at a concentration of 100 µM, a more substantial upregulation was observed. However, at concentrations up to 500 µM, only a moderate increase was observed, and we were concerned about potential cytotoxic effects. Therefore, we chose to continue our investigations with a concentration of 100 µM (Figure 1A).

### 3.2. Time Course Studies Revealed a Sustained Linear Increase in HIF-1α Expression

After optimizing the dosage, we performed a time course study at the selected optimal concentration of 100 µM roxadustat. This study involved examining the expression of nuclear HIF-1α protein at 1-, 2-, 6-, and 24 h post-treatment. The results showed that there was a nearly linear increase in HIF-1α expression from 1 to 24 h. Initially, HIF-1α levels showed upregulation at the 1-h mark (fold change of 1.54) and then continued to increase steadily over 24 h to a maximum 5.85-fold increase. This suggests a sustained activation of related signaling pathways (Figure 1B).

### 3.3. Roxadustat Upregulated HIF-1α Expression 4 Hours Post-Treatment in Mice

**Figure 2:**
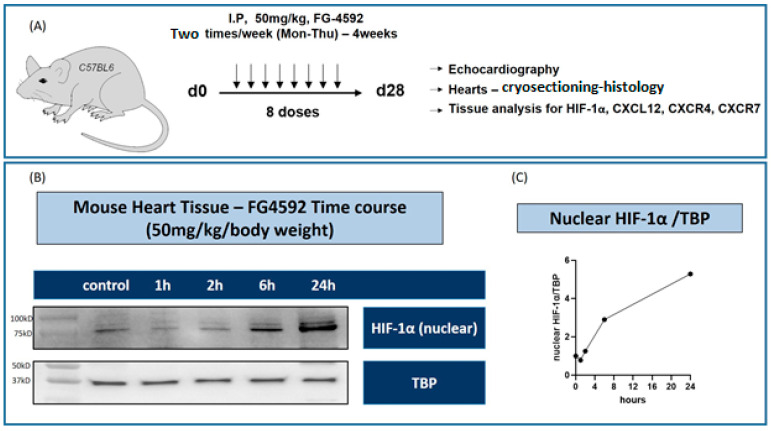
(**A**) Experimental design. (**B**) In vivo time course of whole heart tissue samples 1–24 h after the application of 50 mg/kg roxadustat. (**C**) Nuclear HIF-1α expression increases notably after 6 h and further rises until 24 h. Experiments were repeated 3 times.

To elucidate temporal changes in nuclear HIF-1α expression following PHI treatment in vivo, C57BL/6 mice were administered a single dose of 50 mg/kg roxadustat. Tissue samples were collected at baseline, as well as 1-, 2-, 6-, and 24 h post-treatment. Western blot analyses were performed to quantify levels of nuclear HIF-1α protein expression (n = 3). As shown in Figure 2B, HIF-1α expression was relatively stable at 1 and 2 h post-treatment compared with the baseline. However, a pronounced 2.9-fold upregulation in HIF-1α expression was observed at 6 h post-treatment. The fold change in HIF-1α expression continued to grow, reaching a 5.3-fold peak at 24 h post-treatment (Figure 2B).

### 3.4. Roxadustat Revealed Significant Upregulation of HIF-1α Target Genes after MI In Vivo

Since we noticed a robust upregulation of nuclear HIF-1α after roxadustat treatment, we proceeded to analyze the transcriptional activity of HIF-1α-related target genes after roxadustat treatment ± MI. Therefore, we quantified mRNA levels of seven known HIF-1α target genes including CXCL12, CXCR4, ACKR3, Vascular Endothelial Growth Factor A (VEGFA), Vascular Endothelial Growth Factor D (VEGFD), Adrenomedullin (ADM), Fibroblast Growth Factor 2 (FGF2), Glucose Transporter 1 (GLUT-1), Collagen Type I Alpha 1 Chain (COL1A1), and Collagen Type VIII Alpha 1 Chain (COL8A1) using quantitative real-time PCR (qPCR). All mRNA levels were normalized to Ribosomal Protein L32 (RPL32), a consistent housekeeping gene, and the results were presented as fold changes relative to the lowest expressing sample within the placebo group.

**Figure 3:**
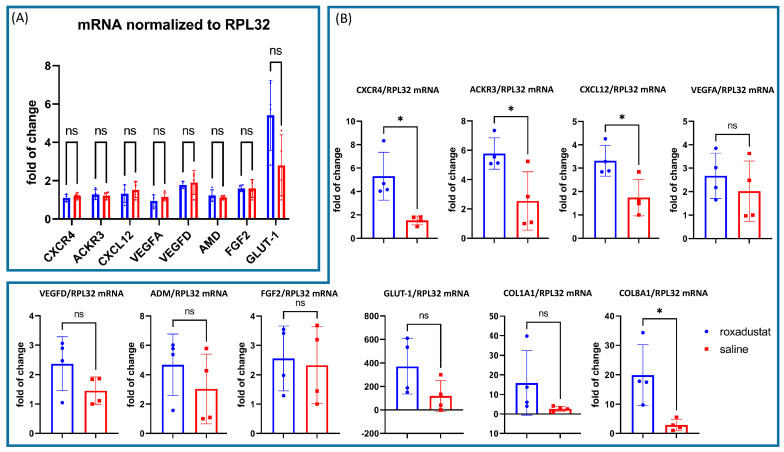
Quantitative PCR of selected HIF-1 regulated genes seven days after roxadustat treatment (n = 4) compared to placebo controls (n = 4) normalized to RPL32. (**A**) We did not observe any changes in mRNA expression after seven days of roxadustat treatment in the absence of myocardial infarction. (**B**) Seven days after MI and roxadustat treatment, we observed a substantial upregulation of CXCL12, CXCR4, ACKR3, and COL8A1 mRNA. All data represent mean ± SD. *p*-values: ns > 0.05; * ≤ 0.05.

We did not find any significant changes in the complete set of genes investigated when roxadustat was administered in the absence of myocardial ischemia (Figure 3A). However, when mice were treated with roxadustat in the presence of myocardial ischemia, we observed a significant increase in mRNA levels for CXCL12, CXCR4, and ACKR3. The fold change in CXCL12 was 3.31 ± 0.66 vs. 1.74 ± 078 (*p* = 0.022), in ACKR3 was 5.78 ± 1.07 vs. 2.54 ± 1.99 (*p* = 0.029), and in CXCR4 was 5.30 ± 2.05 vs. 1.53 ± 0.38 (*p* = 0.011) when comparing the roxadustat-treated group (n = 4) to the control group (n = 4).

There was also a noticeable increase in collagen mRNA. While the COL1A1 mRNA increase was not significant (15.90 ± 16.47 vs. 2.65 ± 1.29; *p* = 0.206) because of the absurdly high SD, there was a clear increase in COL8A1 mRNA (19.85 ± 10.34 vs. 2.85 ± 1.95; *p* = 0.018). The other genes investigated like VEGFA (2.76 ± 1.00 vs. 2.08 ± 1.32 *p* = 0.447), VEGFD (2.13 ± 0.82 vs. 1.31 ± 0.42 *p* = 0.126), ADM (4.68 ± 2.09 vs. 3.03 ± 2.38; *p* = 0.339), and FGF2 (2.56 ± 1.10 vs. 2.33 ± 1.31; *p* = 0.799) showed numerical higher mRNA expression levels without reaching statistical significance (Figure 3B).

### 3.5. Western Blot Analysis Reveals Upregulated Chemokine Receptor Expression 7 Days Post-MI

Next, we investigated whether roxadustat could upregulate the HIF-1α target genes CXCL12, its receptor CXCR4, and ACKR3 on the protein level. We performed Western blot analyses on whole heart tissue samples collected from mice 7 days post-MI. The mice were divided into two groups as follows: a roxadustat-treated group (n = 3) that received 50 mg/kg roxadustat and a placebo group (n = 3) that was given a saline solution.

**Figure 4:**
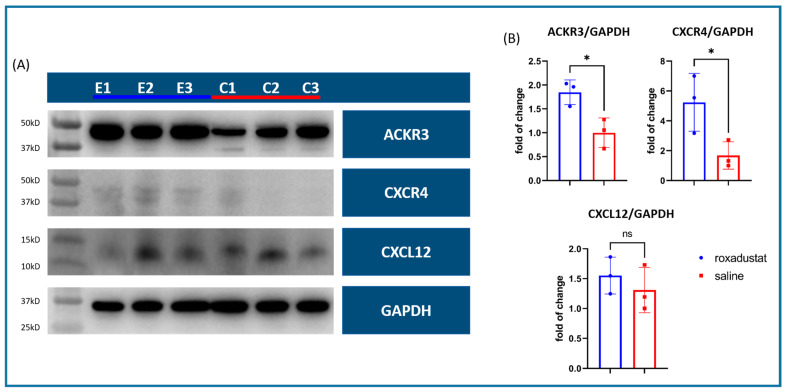
Western blots of whole heart tissue samples 7 days after LAD ligation. (**A**) Roxadustat-treated (E1–3; n = 3) vs. placebo-treated control (C1–3; n = 3) mice protein expression of ACKR3, CXCR4, CXCL12, and glyceraldehyde-3-phosphate dehydrogenase (GAPDH) as a loading control. (**B**) ACKR3 and CXCR4 were significantly higher expressed in the roxadustat-treated group, while CXCL12 was only slightly elevated. All data represent mean ± SD. *p*-values: ns > 0.05; * ≤ 0.05.

The data obtained from this study showed that the protein expression level of CXCL12 was slightly elevated in the roxadustat-treated group, but the increase was not statistically significant (1.55 ± 0.31 vs. 1.31 ± 0.38; *p* = 0.435). However, we found that both CXCR4 and ACKR3 receptors were significantly upregulated in the treated group. Densitometric analysis showed an increase in CXCR4 (5.24 ± 1.94 vs. 1.68 ± 0.91; *p* = 0.045) and ACKR3 (4.30 ± 0.95 vs. 2.05 ± 0.94; *p* = 0.044) in the treated group compared with the placebo group (Figure 4).

### 3.6. Increased CD 206+ M2-Macrophage-Associated Cells after Roxadustat Treatment

**Figure 5:**
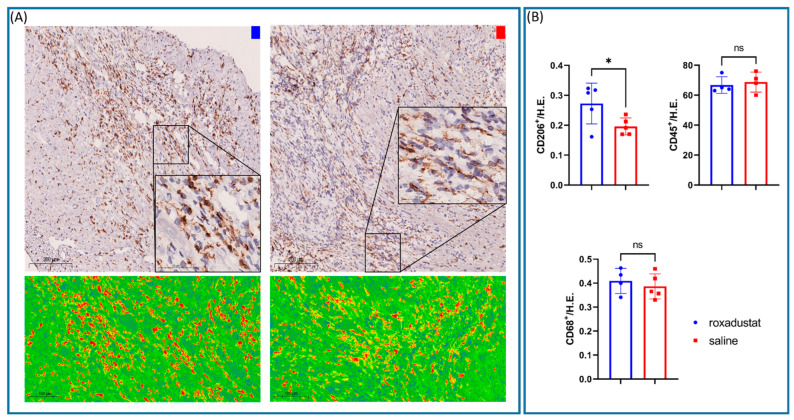
(**A**) M2 macrophage CD 206+ staining of heart tissue from roxadustat-treated and placebo mice, with a heat map indicating expression levels. (**B**) The accompanying bar graphs compare the quantities of immune cell markers CD45, CD68, and CD206 between the roxadusat-treated and placebo-treated groups, reflecting changes in leukocyte infiltration and macrophage activity post-treatment. All data represent mean ± SD. *p*-values: ns > 0.05; * ≤ 0.05.

We conducted immunohistochemistry on heart tissue sections from mice 7 days after myocardial infarction to understand the cellular changes associated with PHI treatment during recovery. We specifically examined the presence of CD 206+ M2 macrophage cells, leucocyte CD 45+ cells, and total macrophage CD 68+ cells. The results of the immunohistochemical analysis showed that there was a significant increase in the number of CD 206+ cells in the group that received roxadustat treatment (n = 5) compared with the group that received a placebo (n = 5) (6.8 ± 3.0% vs. 2.8 ± 1.3%; *p* = 0.049). However, there was no significant difference in the number of CD 68+ (5.3 ± 2.6% vs. 5.2 ± 2.3%; *p* = 0.531) and CD 45+ (66.8 ± 5.5% vs. 68.8 ± 6.7%; *p* = 0.662) cells between the roxadusat-treated and placebo-treated groups (Figure 5).

### 3.7. Immunofluorescence Analysis of the Apoptotic Index

**Figure 6:**
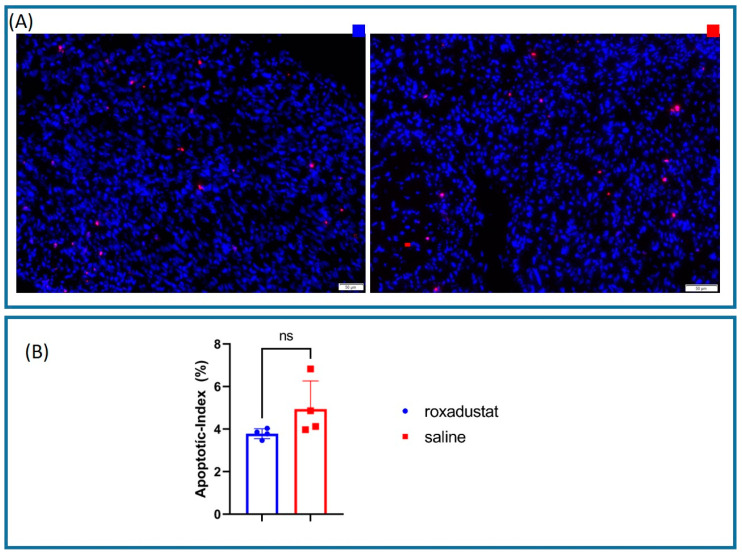
(**A**) Immunofluorescence images of roxadustat-(top left) and saline-(top right) treated sections were fixed and stained for DAPI (blue) or TUNEL (red). (**B**) The percentage difference in apoptotic index between the roxadustat and saline groups did not exhibit a significant difference. All data represent mean ± SD. *p*-values: ns > 0.05.

To quantify the extent of apoptosis in the murine heart sections, we used an immunofluorescent terminal deoxynucleotidyl transferase dUTP Nick-End Labeling (TUNEL) assay, detecting DNA fragmentation resulting from apoptotic signaling (Figure 6A).

Upon quantification of the apoptotic index, defined as the percentage of TUNEL-positive cells relative to the total cell count, we noted a non-significant trend towards a decreased apoptotic index in roxadustat-treated (n = 4) compared to placebo-treated (n = 4) mice (3.78 ± 0.235% vs. 4.95 ± 1.32%; *p* = 0.113) (Figure 6B).

### 3.8. Echocardiographic Assessment Revealed Significant Improvements in Multiple Cardiac Parameters in Roxadustat-Treated Mice 28 Days Post-Myocardial Infarction

To comprehensively evaluate cardiac function following roxadustat treatment in vivo, echocardiographic measurements were performed on mice 28 days after the onset of myocardial infarction. The roxadustat-treated (n = 10) and saline-treated placebo groups (n = 10) were compared by blinded investigators across a range of parameters.

**Figure 7:**
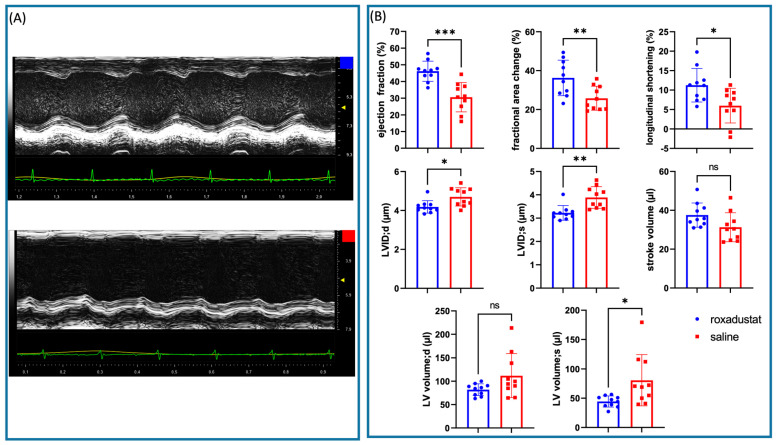
Echocardiographic measurements. (**A**) M-Mode of the LV cavity in the parasternal long axis view on a mid-ventricular level (blue = roxadustat-treated mice; red = placebo mice). (**B**) Echocardiographic measurements comparing roxadustat-treated (n = 10; blue) and saline-treated infarcted mice (n = 10; red). All data represent mean ± SD. *p*-values: ns > 0.05; * ≤ 0.05 ** ≤ 0.01; *** ≤ 0.001.

As shown in Figure 7A,B, ejection fraction (EF) was significantly higher in the roxadustat-treated group compared with the placebo group (46.14 ± 6.03% vs. 30.57 ± 8.80%; *p* < 0.001). The fractional area change (FAC) was also significantly improved in the treated group compared with the placebo (36.27 ± 9.12 vs. 25.79 ± 6.17; *p* = 0.008).

Left ventricular internal diameter during diastole (LVEDD) and systole (LVEDS) also revealed a significantly improved left ventricular (LV) remodeling in favor of roxadustat treatment (4.18 mm ± 0.32 mm vs. 4.7 ± 0.47 mm; *p* = 0.011 and 3.23 ± 0.31 vs. 3.88 ± 0.46; *p* = 0.002). Longitudinal shortening (LS) was significantly higher in the roxadustat-treated group compared with the placebo group (11.26 ± 4.32% vs. 6.00 ± 4.47%; *p* = 0.015).

Systolic LV volume was significantly lowered in the roxadustat-treated group compared with the placebo group (44.42 ± 9.52 µL vs. 80.55 ± 43.70 µL, *p* = 0.029). In the contrary, LV volume during diastole was also lower in the roxadustat-treated group but did not reach significance compared with the placebo group (81.97 ± 12.58 µL vs. 111.83 ± 46.96 µL; *p* = 0.080). Therefore, stroke volume [19] was also not significantly different between the groups, with a mean of 31.29 ± 8.63 µL in the treated group and 24.48 ± 5.52 µL in the placebo group (*p* = 0.056).

In summary, the echocardiographic data indicate that roxadustat significantly improved multiple cardiac parameters, including EF, FAC, LVEDD, LFS, and LV volumes, while LVEDD and SV remained unchanged. These findings suggest that HIF upregulation via roxadustat has a beneficial impact on cardiac function and LV remodeling 28 days post-myocardial infarction (Figure 7B).

**Figure 8:**
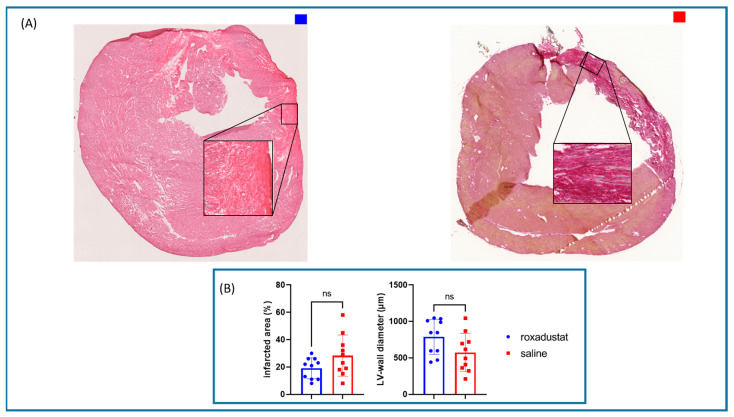
(**A**) Picro-Sirus Red-stained heart sections. (**B**) Bar graphs representing infarct area and LV wall diameter (n = 10; blue bars) and placebo-treated mice (n = 10; red bars). All data represent mean ± SD; ns not significant.

Although the measurements of infarcted area size in Picro-Sirius Red-stained sections did not show significant difference between the roxadustat-treated group (n = 10) and the placebo group (n = 10), there was a trend towards a reduced infarct size in the roxadustat-treated mice compared with the placebo-treated mice (19.1 ± 7.9% vs. 28.4 ± 15.2%; *p* = 0.102, Figure 8A,B).

We also assessed the thickness of the myocardium in the infarcted region as an additional measure of tissue repair and remodeling. Again, we did not observe a significant difference. However, the average myocardial thickness in the infarcted region was 788 ± 240 µm for the roxadustat-treated mice and 575 ± 262 µm for the saline-treated mice (*p* = 0.073, Figure 8A,B).

## 4. Discussion

In this study, we investigated the therapeutic potential of the clinically approved PHI, roxadustat, for use as a therapy after MI in mice. Our findings indicate a substantial upregulation of nuclear HIF-1 both in vitro and in vivo, which was linked to an increase in the expression of HIF-1α target genes from the CXCL12/CXCR4/ACKR3 chemokine axis. This increase was associated with a higher number of CD206+ reparative M2-like macrophages compared with total CD68+ macrophage cells. As a result, there was an improvement in cardiac function and a reduction in negative changes following myocardial ischemia (as shown in the graphical abstract).

HIF-1α is a crucial transcription factor in the cardiovascular system [20], as it regulates gene expression of the CXCL12/CXCR4/ACKR3 axis during ischemia [21,22]. Previous research by our group showed that DMOG, an unspecific prolyl-hydroxylase inhibitor, can upregulate nuclear HIF-1α and the chemokine CXCL12 in the mouse heart after MI, leading to improved cardiac repair [23]. Other studies have also revealed that preconditioning with DMOG can cardio-protect hearts from ischemic damage [24]. However, DMOG has far too many side effects for use in humans [25]. Approved by the EMA in 2021, roxadustat has advantages over traditional treatments with erythropoietin-stimulating agents as it more accurately replicates a physiological state of hypoxemia, which also regulates iron beneficially in managing renal anemia in patients bearing CKD [26]. However, the effects of roxadustat after myocardial ischemia have not been investigated yet. 

Our data revealed that the administration of roxadustat after myocardial ischemia can increase the expression of HIF-1α and upregulate HIF-1α-related target genes, which was associated with the attenuation of adverse remodeling. These findings indicate the potential use of roxadustat in clinical practice. A study conducted by Deguchi et al. also demonstrated that pretreatment with roxadustat can protect against myocardial I/R injury in mice [27].

Intriguingly, our research revealed a nuanced orchestration of the inflammatory response post-myocardial ischemia, likely modulated by HIF-1. We observed an increase in the number of reparative CD206+ cells, while the number of total CD68+ macrophage cells remained the same. This suggests that there was an increase in the proportion of reparative M2 macrophages, which is in line with previous findings for more toxic PHI inhibitors like those outlined in [11]. M2 macrophages are known for their release of anti-inflammatory cytokines and growth factors, extracellular matrix deposition, and clearance of debris, while M1 macrophages are involved in pathogen killing and initiating an immune response [28].

Extensive research has shown that hypoxia-inducible factor plays and important role in mitigating apoptosis during ischemic conditions [11,29]. Our study revealed a marked trend towards reduced apoptosis rates in roxadustat-treated mouse hearts, although the results did not attain statistical significance. This discrepancy may be attributable to the low sample size and high variability in apoptosis rates observed in the placebo group, in contrast to the minimal variability in the roxadustat-treated cohort. These observations are congruent with clinical data suggesting that similar coronary pathologies do not necessarily result in comparable clinical outcomes. Notably, the treated group exhibited a more uniform response, suggesting that HIF-based therapy may have the potential to normalize individual variations in disease manifestation.

During our investigation, we observed a trend towards smaller infarct areas and a thicker left ventricular wall in the roxadustat-treated mouse hearts. However, the high standard deviation in the placebo group, given this group size, made it difficult to reach a significant difference, while the variation in the treated samples was relatively small. This observation highlights the effectiveness of roxadustat treatment, as it suggests a uniform response across the treated group, and indicates that roxadustat may specifically compensate for individual disadvantages. We also observed an interesting qualitative trend during our investigation. Certain hearts that appeared less infarcted when harvested after 7 days of treatment had large pale areas but were not markedly thinned. Surprisingly, an unexpected trend was noted: these samples seemed to have a much higher degree of collagen mRNA expression relative to others that presented more pronounced infarction characteristics. This could be consistent with the observation that echocardiographically, two animals in the placebo group developed a pronounced apex aneurysm after MI, resulting in prolongation of the LV in systole (Figure 7B, longitudinal shortening). While this introduces a notable point of discussion, it is crucial to interpret these observations cautiously. Because of the limited sample size and the absence of quantifiable data to substantiate these findings, we recognize that these are preliminary results. Additionally, these results suggest the possibility that individual biological variations may specifically influence the myocardial response within the HIF-collagen pathway. This hypothesis requires further investigation in studies with larger cohorts.

Based on our observation, we hypothesize that the increased collagen synthesis and scar deposition induced by roxadustat provide sufficient stability to prevent dilation and adverse remodeling. However, this should also be reflected in the wall thickness. In our study, we divided the infarcted area into five equal-sized segments and consistently measured the wall thickness at the borderlines of these segments, aiming for a systematic and replicable method. This approach may not always measure the thinnest part of the wall, and a different method could potentially yield more fitting results.

Our attention was focused on HIF-1 downstream targets, specifically the CXCL12/CXCR4/ACKR3 axis, renowned for its significant role in cardiac repair and development. Previous studies have predominantly emphasized CXCL12 interactions with CXCR4, responsible for progenitor cell recruitment and neoangiogenesis. However, recent findings also pointed to a pivotal role for ACKR3, not merely as a scavenger receptor but also as a significant contributor to cardiac repair via β-arrestin-mediated signaling pathways [6,7,8,9,10,11]. The upregulation of CXCL12, CXCR4, and, particularly, ACKR3, in the roxadustat-treated specimens aligns well with this hypothesis. Notably, the prominence of ACKR3 expression, as noted by Ishizuka et al., in conjunction with detrimental cardiac outcomes in ACKR3-deficient models, consolidates its importance in mitigating pathological remodeling [9]. This suggests that the benefits of roxadustat may be regulated by direct HIF modulation, extending to ACKR3-mediated pathways. Although we predominantly focused on the infarcted site, we acknowledge the potential compensatory alterations in the non-infarcted myocardium. This aspect was unexplored in this study and could provide additional insights into the overall cardiac response to injury.

Research conducted by Levent et al. using engineered human myocardium showed the effectiveness of roxadustat in mitigating hypoxia/reoxygenation injury. Importantly, their findings demonstrated that roxadustat not only prevents contractile failure but also significantly reduces apoptosis in engineered human tissue, which is in line with our findings [29]. This revelation is particularly crucial given the complexities of replicating human cardiac pathology in animal models.

Considering these insights from engineered human tissue, which mirror our observations, and those from other animal model studies, we believe that our data further high-light the translational potential of PHIs in myocardial infarction therapy. The combined evidence strongly supports the therapeutic use of roxadustat in real-world clinical scenarios. A recently published study by Yazaki et al. in patients with HF and CKD showed that the PHI vadadustat led to improvements in anemia and symptoms of HF, also arguing for the use of PHI in clinical practice [30]. Another study is currently investigating the effect and safety of roxadustat in the treatment of type 4 cardiorenal syndrome [31]. 

However, the temporal aspect of this treatment necessitates further exploration, given the non-discriminative activation of both HIF-1 and HIF-2 by PHIs. While short-term upregulation of collagen synthesis via HIF-1 is seemingly advantageous, its prolonged activation could be disadvantageous. In line with this notion, Ikeda et al. presented findings suggesting that an excessive and prolonged elevation of HIF-1 could potentially culminate in cardiac rupture, a process thought to be mediated by another HIF-1 target gene, tumor protein p53 [32].

These observations underscore the imperative for a carefully balanced approach to HIF modulation in therapeutic strategies. Future investigations should aim to elucidate the optimal therapeutic window to maximize reparative benefits while averting harmful consequences. However, it is important to note that an initial treatment, even before reaching the catheter lab, appears to be important to exploit the protective effects fully.

In our study, we conducted in vivo experiments where we administered a dosage of 50 mg of roxadustat per kg of body weight. The dose used in this mouse model corresponds to a human equivalent dose of approx. 4 mg/kg, which is 1.6 times higher than the dose of 2.5 mg/kg used in clinical trials in humans [33,34]. In a clinical setting, it might be important to tailor dosages according to individual hemoglobin levels since it is known that iron deficiency worsens symptoms of heart failure. Patients without anemia may necessitate lower dosages, and potential side effects such as hypertension and thrombosis have to be considered.

## 5. Conclusions

To summarize, our data strongly suggest that roxadustat, a PHI that is clinically approved, shows great promise in reducing adverse remodeling after myocardial ischemia, preventing loss of cardiac function, and potentially repairing damaged heart tissue.

## Data Availability

All data is available from the corresponding author upon request.
